# In Vitro Evaluation of Poly(D,L-lactide-co-glycolide) In Situ Gels and Pharmacokinetics Following Subcutaneous Injection in Rats for Model Drugs

**DOI:** 10.3390/pharmaceutics18020219

**Published:** 2026-02-09

**Authors:** Sandy Van Hemelryck, Charlotte Vercammen, Eline Seldeslachts, Koen Wuyts, Ils Pijpers, René Holm, Erik Mannaert, Peter Langguth

**Affiliations:** 1 Clinical Pharmacology and Pharmacometrics, Johnson & Johnson, Turnhoutseweg 30, 2340 Beerse, Belgium; 2 Faculty of Pharmaceutical Sciences, University of Leuven, Herestraat 49, 3000 Leuven, Belgium; 3 Preclinical Safety and Translational Sciences, Johnson & Johnson, Turnhoutseweg 30, 2340 Beerse, Belgium; 4 Department of Physics, Chemistry and Pharmacy, University of Southern Denmark, Campusvej 55, 5230 Odense, Denmark; 5 Department of Biopharmaceutics and Pharmaceutical Technology, Johannes Gutenberg University, Staudingerweg 5, 55128 Mainz, Germany

**Keywords:** in situ gels, solubility, in vitro release, pharmacokinetics, sustained release, in vitro–in vivo correlation

## Abstract

**Background/Objectives.** This research supports the development of long-acting injectables (LAIs) via in situ gel (ISG) technology by illustrating the influence of drug properties and formulation variables on in vitro drug release (Part 1), and providing an example of a point-to-point in vitro–in vivo correlation (IVIVC) for celecoxib ISGs (Part 2). **Methods/Results.** Part 1 evaluated the in vitro release (IVR) for ISGs containing 10 mg/g of five model drugs—paracetamol, theophylline, felbinac, indomethacin, and celecoxib—using two different poly(D,L-lactide-co-glycolide) (PLGA) grades with lactide/glycolide ratios (L/G) of 50:50 or 85:15 in N-methyl-2-pyrrolidone (NMP) at polymer/solvent ratios of 30/70% or 40/60% (*w*/*w*). The results demonstrated sustained IVR, with approximately 80% of the drug released within 1 to 5 days for the sparingly soluble compounds paracetamol and theophylline ISGs, and within 1.5 to 11 days, 3 to over 20 days, and 19 to 74 days for the slightly soluble compounds felbinac, indomethacin, and celecoxib, respectively. The IVR rate increased with decreasing polymer lipophilicity and concentration and with increasing drug solubility in the IVR medium. In Part 2, the pharmacokinetics of celecoxib ISGs were assessed following subcutaneous (SC) injection in rats. A point-to-point IVIVC was established between the fraction of drug absorbed derived via deconvolution (deconvoluted *F*_abs_) and the fraction dissolved (observed *F*_diss_) obtained in Part 1, based on Korsmeyer–Peppas fitting and release phase-specific scaling. **Conclusions.** In summary, this research highlights the significant impact of drug solubility, polymer grade, and concentration on the IVR rates of ISGs and provides an example of a point-to-point IVIVC for celecoxib ISGs with varying polymer concentrations and grades, following SC injection in rats.

## 1. Introduction

Chronic diseases, such as cardio- or cerebrovascular disorders, cancer, chronic respiratory diseases, and diabetes, are the leading cause of death worldwide [1]. Despite the availability of effective therapies, disease management often remains inadequate, primarily due to poor adherence and long-term persistence with prescribed treatments.

Long-acting injectables (LAIs) have emerged as a promising strategy to enhance patient compliance through extended-release drug delivery systems. These formulations can provide sustained drug exposure for weeks to months with a single administration. Furthermore, LAIs can be engineered to tailor drug release profiles to minimize adverse effects and enable targeted delivery. Additionally, they offer advantages for compounds with limited oral bioavailability, high first-pass metabolism, or susceptibility to gastrointestinal degradation.

Commonly applied LAI technologies encompass aqueous suspensions, polymeric microspheres, oily solutions, and implants. However, these platforms exhibit inherent limitations: aqueous suspensions and oily solutions are typically limited to drugs with low aqueous or high lipid solubility, respectively; microsphere manufacturing involves complex and costly processes; and preformed implants require invasive procedures, including surgery. Recently, in situ gels (ISGs), which transition from a liquid to a gel or solid state upon administration, have gained increasing interest for LAI applications. These systems utilize polymers solubilized in organic solvents or aqueous media, which solidify in response to physiological stimuli (e.g., temperature, pH), chemical reactions (e.g., polymerization), or solvent exchange. The resulting matrix modulates drug release kinetics.

Most ISGs are developed using biodegradable co-polymers of lactide and/or glycolide, such as poly(D,L-lactide) (PLA) and poly(D,L-lactide-co-glycolide) (PLGA), which degrade via hydrolysis in an aqueous environment. Hydrolysis involves the cleavage of covalent ester bonds, facilitated by water, resulting in a decrease in molecular weight, destabilization of the polymer matrix, and eventual solubilization of the polymer fragments [2,3,4]. FDA-approved ISGs include Atridox (doxycycline, poly(D,L-lactide) (PLA) in N-methylpyrolidone (NMP)), Camcevi (leuprolide, PLA in NMP), Eligard (leuprolide, poly(D,L-lactide-co-glycolide) (PLGA85:15, 75:25, 50:50) in NMP), Perseris (risperidone, PLGA80:20 in NMP), Sublocade (buprenorphine, PLGA50:50 in NMP), and Uzedy (risperidone, methoxy-poly(ethylene glycol)-co-PLA (mPEG-PLA), PLA-PEG-PLA in dimethylsulfoxide (DMSO)), with administration intervals ranging from 1 week to 6 months [5].

The advantages of PL(G)A-based ISGs include their relatively straightforward manufacturing process, minimally invasive administration via intramuscular or subcutaneous injection, and the capacity to modulate drug release through polymer selection and formulation parameters. Formulation studies demonstrated that increasing polymer concentration, lipophilicity (e.g., higher lactide/glycolide (L/G) ratios, lipophilic end groups), or molecular weight generally slows drug release by reducing erosion rate, swelling, and gel porosity [6,7,8,9]. However, the influence of drug physicochemical properties—including solubility, molecular weight, lipophilicity, and solid state characteristics—on release kinetics remains poorly understood [10].

In vitro release (IVR) testing serves as a valuable tool for characterizing drug release, guiding drug development and supporting manufacturing and formulation modifications. Nevertheless, the complex interplay of mechanisms, including phase inversion, diffusion, polymer relaxation, erosion and swelling, along with discrepancies between in vitro and in vivo drug release behaviors complicates the establishment of robust in vitro–in vivo correlations (IVIVCs) [11,12]. Drug release often exhibits multiphasic kinetics, characterized by an initial burst driven by rapid surface diffusion during phase inversion, a subsequent slower phase governed by bulk diffusion and polymer relaxation post-solidification, and an eventual accelerated phase resulting from polymer erosion and dissolution. Although several point-to-point Level A IVIVCs have been reported for PLGA ISGs or microspheres with minor variations in formulation or manufacturing process, model validation remains a significant challenge [13,14,15]. The variability of the in vitro–in vivo relationship across different phases diminishes the predictive accuracy of time-scaled empirical models such as Hill, Weibull, or Makoid–Banakar, which either overestimate or underestimate the maximal plasma concentration (C_max_). Recently, semi-mechanistic kinetic models, including Higuchi (*C*(*t*) = *K* × *t*^0.5^), Korsmeyer–Peppas (*C*(*t*) = *K* × *t^n^*), and Peppas–Sahlin (*C*(*t*) = *K*_1_ × *t^n^*^1^ + *K*_2_ × *t^n^*^2^), have been applied to analyze drug release from polymer-based systems such as ISGs [16,17,18]. The Higuchi model describes Fickian drug diffusion, relating drug release to the square root of time, while the Korsmeyer–Peppas and Peppas–Sahlin models incorporate flexible exponents *n* to provide mechanistic insights. Specifically, *n* ≤ 0.5 indicates (pseudo-)Fickian-type diffusion, 0.5 > *n* < 1 suggests anomalous, non-Fickian release involving both diffusion and matrix relaxation, *n* = 1 corresponds to case II (zero order) release, and *n* > 1 signifies super-case II transport driven by polymer erosion, swelling, and/or dissolution [19,20,21,22,23,24]. Identifying factors that influence drug release and the in vitro–in vivo relationship, in conjunction with mechanistic kinetic modeling, are essential in the development of reliable IVIVCs for these complex ISGs.

This research consisted of two parts. The objective of Part 1 was to investigate the influence of drug solubility on release kinetics in ISGs and to assess the capacity of ISGs to modulate the release of water-soluble synthetic drugs, thereby establishing their potential as a platform for achieving release durations of weeks to months. Accordingly, the IVR profiles of five model drugs—paracetamol, theophylline, felbinac, indomethacin, and celecoxib—were evaluated in ISGs formulated with various PLGA grades and concentrations. These drugs were selected to span a wide range of aqueous solubilities, with molecular weights < 500 g/mol and logP values < 5 (see Table 1). All compounds exhibit high solubility in N-methyl pyrrolidone (NMP) and have been reported as safe and well tolerated following subcutaneous (SC) or intramuscular (IM) injection in rat models. A drug concentration of 10 mg/g was chosen for all ISGs to ensure complete dissolution of all compounds within the formulations (see Table 1), allowing for direct comparison across compounds to be made without confounding effects from undissolved or suspended drug.

Polymer grades and concentrations were chosen based on FDA-approved ISGs to achieve controlled release durations from several weeks to months, while maintaining acceptable syringeability. The objective of Part 2 was to establish a point-to-point IVIVC for celecoxib ISGs following subcutaneous injection in rats, based on kinetic drug release modeling. Celecoxib was selected for this purpose because its IVR profiles, generated in Part 1, demonstrated distinct differences across formulations and were monitored until complete IVR was achieved.

## 2. Materials and Methods

### 2.1. Materials

Paracetamol, theophylline, felbinac, indomethacin, and celecoxib were purchased from Glentham Life Sciences Ltd. (Corsham, UK); the physicochemical properties are summarized in Table 1 [25]. NMP and PLGA were obtained from Asland (Covington, KY, USA) as Pharmasolve, PLGA Viatel DLG 5003A (L/G ratio 50:50, acid-terminated, inherent viscosity 0.33 dL/g, referred to as PLGA50:50) and PLGA Viatel DLG 8503A (L/G ratio 85:15, acid-terminated, inherent viscosity 0.36 dL/g, referred to as PLGA85:15). Polyethylene glycol 400 (PEG400) was sourced from Clariant (Frankfurt am Main, Germany). Water for injections was ordered from Sterop Laboratoria (Brussels, Belgium) or Baxter (Lessines, Belgium). All other chemicals were purchased as reagent grade from commercial sources and used as received.

### 2.2. Formulation Preparations

Table 2 summarized the formulations prepared for the in vitro and/or in vivo assessments conducted in this study.

ISG formulations 1, 2, 3, and 4, each containing 10 mg/g of the model drug, were formulated in PLGA50:50/NMP and PLGA85:15/NMP at 30/70% (*w*/*w*) as well as 40/60% (*w*/*w*). The preparation involved dissolving the polymer in NMP, followed by the addition of the model drug, with the mixture shaken at 100 rpm in an Innova incubator shaker (New Brunswick Scientific, Edison, NJ, USA) at 25 °C until a clear solution was obtained.

Additionally, formulation 5, containing 1 mg/mL celecoxib in PEG400/water 70:30% (*v*/*v*), was prepared by dissolving the drug in PEG400 and subsequently diluting with water.

For formulations intended for rat administration, sterile filtration through a 0.2 µm sterile Millex polyvinylidene fluoride (PVDF) filter (Merck KGaA, Darmstadt, Germany) was performed to ensure sterility.

### 2.3. In Vitro Release for In Situ Gels Containing Model Drugs

#### 2.3.1. In Vitro Release Studies

The IVR profiles of ISG formulations 1 to 4 containing 5 model drugs were assessed in quadruplicate in 100 mL glass vials containing 50 mL 0.05 M phosphate buffer pH 7.4. For celecoxib ISGs, the phosphate buffer was supplemented with 3% (*w*/*v*) sodium lauryl sulphate (SLS) to enhance solubility. The dissolution set-up was designed to simulate the physiological environment and ensure sink conditions, based on experimentally determined solubility data included in Table 1, for all model drugs. The vials were incubated in an Innova incubator shaker at 37 °C on a plate shaking at 90 or 100 rpm. In each vial, 0.3 g of the respective formulation was injected using a syringe. At predetermined time points (ranging from 1 to 480 h for paracetamol, theophylline, felbinac, and indomethacin, and up to 2568 h for celecoxib), 4 mL of dissolution medium was sampled using a syringe with needle and then replaced with an equal volume of fresh buffer maintained at the same temperature. The collected samples were filtered through a Spartan syringe filter (0.2 µm, 30 mm; Cytiva, Maidstone, UK) and stored at room temperature until analysis (see Section 2.3.2). Based on the quantification of the model drugs in the dissolution medium, the percentage of drug dissolved and the fraction of drug dissolved (observed *F*_diss_ = percentage drug dissolved/100%, normalized to 1 in case of complete drug release) were calculated. To further summarize the observed IVR profiles, the time to reach 50% and 80% of dissolved drug was derived via linear interpolation between the observed IVR data. Statistical comparisons of IVR data across ISGs were based on a two-tailed, heteroscedastic *t*-test [26] available in Microsoft Excel and the calculation of difference (*f*1) and similarity factors (*f*2) for the mean profiles of *N* = 4 per formulation [27] using Microsoft Excel.

$$f1=\frac{\sum_{t=1}^{n}\left|R_{t}-T_{t}\right|}{\sum_{t=1}^{n}R_{t}}\times 100$$

$$f2=50\times log\left\{{\left[1+\frac{1}{n}\sum_{t=1}^{n}{\left(R_{t}-T_{t}\right)}^{2}\right]}^{-0.5}\times 100\right\}$$

where *n* is the number of time points, and *R_t_* and *T_t_* are the mean percent dissolved of the reference and test product at time *t*, respectively.

#### 2.3.2. Analytical Method for In Vitro Release Studies

The quantification of model drugs in the IVR samples was performed using an Acquity H-Class ultra-high-performance liquid chromatography (UHPLC) system equipped with an Acquity CSH C18 column (1.7 µm, 2.5 × 50 mm), a photodiode array (PDA) detector, and Empower 3 software for data acquisition and analysis (Waters Corporation, Milford, MA, USA). The column temperature was maintained at 45 °C and detection was performed at a wavelength of 251 nm. Gradient elution was applied with mobile phases (A) 0.1% (*v*/*v*) trifluoroacetic acid (TFA) in water and (B) acetonitrile (ACN) at a flow rate of 0.8 mL/min. The gradient program involved reducing the percentage of (A) from 95% to 5% over the first 3.5 min, holding at 5% for 0.5 min, increasing back to 95% in 0.5 min, and maintaining at 95% for an additional minute, resulting in a total run time of 5.5 min.

### 2.4. Pharmacokinetic Study in Rats for Celecoxib In Situ Gels

#### 2.4.1. Animals

For the pharmacokinetic study, male Sprague Dawley rats weighing 250 to 350 g (actual body weights at the time of dosing ranged from 248 to 294 g) were delivered by Charles River (Sulzfeld, Germany). The rats were group-housed in polysulfone cages with corn cob bedding material in airconditioned (20–24 °C) rooms under a 12 h light cycle. Aspen wood block (Datesand, Stockport, UK) and Rodent retreat (Bio-Serv, Flemington, NJ, USA) were used as cage enrichment, and food and water were available ad libitum. Before starting the pharmacokinetic study, the rats were acclimatized for at least 4 days.

The study was performed following the guidelines of the Janssen Pharmaceutica Animal Ethics Committee (Beerse, Belgium), the local Belgium laws controlling the use of experimental animals, and the EC Directive 2010/63/EU.

#### 2.4.2. Pharmacokinetics

Twenty rats were divided into 5 groups based on body weight (4 animals per group, as detailed in Table 3) to ensure comparable mean body weights across groups (268 to 270 g). Groups 1 to 4 received SC injections of ISG formulations 1 to 4 at 10 mg/kg in the back, respectively, whereas group 5 received an IV dose of 1 mg/kg PEG400 solution via the vena saphena. All doses in mg/kg were adjusted to each animal’s actual body weight at the time of administration.

Blood samples (32 µL) were collected via tail vein at time points from 1 to 2016 h (ISG formulations 1 to 4) or 1 to 72 h (solution formulation 5) into Vitrex micro hematocrit tubes “soda lime glass” containing potassium ethylenediaminetetraacetic acid (K2.EDTA) (Vitrex Medical A/S, Herlev, Denmark), stored on melting ice and then centrifuged at 1500× *g* and 5 °C for approximately 10 min. Plasma (10 µL) was collected with Vitrex end-to-end pipettes in FluidX tubes (Brooks Life Sciences, Chelmsford, UK) and stored at −20 °C for bioanalysis (see Section 2.4.3).

PhoenixWinNonlin (Certara, Princeton, NJ, USA) was used to calculate celecoxib pharmacokinetic parameters by non-compartmental analysis and fraction absorbed via deconvolution (deconvoluted *F*_abs_, normalized to 1 in case of complete absorption).

Deconvolution function:

*F*_abs_(*t*) = *C*(*t*) \* *C_δ_*(*t*)

Unit impulse response (UIR) function:

*C_δ_*(*t*) = *A* exp[−*αt*] + *B* exp[−*βt*]

where *F*_abs_(*t*): fraction of drug absorbed at time *t*; *C*(*t*): drug concentration at time *t*; \*: Phoenix WinNonlin deconvolution operation; and *C_δ_*(*t*): unit impulse response function with macro-constants *A*, *B*, *α*, and *β*, estimated from the mean pharmacokinetic profile of formulation 5 using a two-compartment, IV-bolus input, first order elimination pharmacokinetic model.

A two-tailed heteroscedastic *t*-test [26] available in Microsoft Excel was applied for statistical comparisons between groups.

#### 2.4.3. Bioanalytical Method for Pharmacokinetics

Bioanalysis was performed using a qualified method developed internally at Johnson & Johnson, demonstrating sufficient robustness, accuracy, and precision for the intended purpose. Rat plasma collected in Vitrex end-to-end pipettes (see Section 2.4.2) was transferred to FluidX tubes by washing out with 100 µL 5% bovine serum albumin (BSA) in phosphate buffer. Then, 22 µL of the washed-out plasma was transferred to another FluidX tube and mixed with 20 µL blank solvent (DMSO), 20 µL internal standard solution (100 ng/mL of 2,5-dimethyl celecoxib in methanol), and 200 µL acetonitrile. Celecoxib calibration standards were prepared in FluidX tubes at concentrations of 1 to 1000 ng/mL by mixing 22 µL blank rat plasma with 5% BSA in phosphate buffer (1:10) with 20 µL spiking solutions of celecoxib in DMSO, 20 µL of internal standard, and 200 µL acetonitrile. All FluidX tubes were placed in a 96-well plate, shaken for 10 min in an orbital shaker, and then centrifuged for 10 min at 5000 to 6000× *g*. The supernatant (diluted with milli-Q water as needed) was analyzed using a liquid chromatography–tandem mass spectrometry (LC-MS/MS) system to determine the levels of celecoxib. The LC-MS/MS system consisted of a Nexera-series high-performance liquid chromatography (HPLC) instrument (Shimadzu Scientific Instruments, Columbia, MD, USA) equipped with an Acquity UPLC BEH C18 column (1.7 µm; 50 × 2.1 mm; Waters Corporation, Milford, MA, USA), maintained at 50 °C, coupled to an API4000 or 5500 triple quadrupole mass spectrometer (AB Sciex, Toronto, ON, Canada) with Turbo Ionspray source operated in the positive ion mode at 400 °C. For HPLC, gradient elution was applied at a flow rate of 0.6 mL/min, mixing mobile phases (A) 0.01 M ammonium carbonate in milliQ water and (B) methanol with a total run time of 3 min: during the first 1.5 min, the (A)/(B) ratio decreased from 50/50% to 2/98%, was kept stable for 0.5 min, was then increased again to 50/50% in 0.1 min, and kept stable for 0.9 min. For MS, multiple reaction monitoring (MRM) was applied with transitions of m/z 379.9 → 316.1 for celecoxib and m/z 393.9 → 330.1 for the 2.5-dimethyl celecoxib internal standard and a collision energy of −28 for celecoxib and −32 for 2.5-dimethyl celecoxib. The celecoxib concentrations of the study samples were calculated by interpolation from a calibration curve. This calibration curve was generated fitting a linear regression model with 1/x^2^ weighting to the peak area ratios of celecoxib to its internal standard plotted versus corresponding celecoxib concentrations.

#### 2.4.4. Clinical Observations

Rats of groups 1 to 5 were checked daily for illness, untoward clinical signs, abnormal behavior or unusual appearance, skin deviations, toxic or pharmacological response, and moribund state or mortality.

### 2.5. Kinetic Drug Release Modeling for In Situ Gels Containing Model Drugs

#### 2.5.1. In Vitro Release for In Situ Gels Containing Model Drugs (Part 1)

IVR profiles obtained for ISGs containing the 5 model drugs were characterized using the Korsmeyer–Peppas model (*C*(*t*) = *K* × *t^n^*), implemented through Microsoft Excel’s Solver add-in to describe drug release kinetics. The model was fitted up to approximately 85% drug release (observed *F*_diss_ ≈ 0.85) for biphasic IVR profiles. For triphasic profiles, fitting was performed using observed *F*_diss_ data up to the final time point of the first phase and from the first time point of the second phase to either the onset of the plateau or the last time point, in cases where no plateau was reached. Since fitted exponents *n* were similar across model drugs and formulations, refitting was performed with *n* fixed at its average value, thereby facilitating the assessment of trends. The goodness of fit of the obtained models was quantitatively evaluated by calculating the Pearson’s correlation coefficient in Microsoft Excel for the entire IVR profile, either up to the first time point where fitted *F*_diss_ exceeded 95% or the last time point if no plateau was observed.

#### 2.5.2. In Vitro–In Vivo Correlation for In Situ Gels Containing Celecoxib (Part 2)

To establish an IVIVC for celecoxib formulations, deconvoluted *F*_abs_ was plotted against observed *F*_diss_ to elucidate the relationship across different release phases. Visual assessment revealed that the initial burst (the first 24 h), slower diffusion, and accelerated phases exhibited distinct in vitro–in vivo relations. While Part 1 involved fitting the combined burst and slower release phase to identify general trends in drug release, Part 2 required segmentation into 3 separate phases, with individual Korsmeyer–Peppas fits for each segment. Subsequently, Microsoft Excel’s Solver add-in was utilized to determine the in vivo scaling factor *A* for each segment, describing the relationship between Korsmeyer–Peppas IVR fits and deconvoluted *F*_abs_ profiles as *C*(*t*) = *A* × *K* × *t^n^*. Consistent scaling trends were identified across formulations and across release phases and re-fitting was performed while holding *A* constant across formulations and phases, where feasible, to avoid overfitting and maximize the utility of the point-to-point IVIVC. Model fit quality was evaluated by determining the Pearson’s correlation coefficient in Microsoft Excel across the full *F*_abs_ profile, either up to the first time point at which *F*_abs_ exceeded 95% or the last time point if no plateau was observed. Phoenix WinNonlin (Certara, Princeton, NJ, USA) was used to convolute modeled *F*_abs_ profiles based on the UIR function estimated from the mean pharmacokinetic profile of formulation 5 (see Section 2.4.2), and to calculate the corresponding pharmacokinetic parameters.

## 3. Results

### 3.1. In Vitro Release for In Situ Gels Containing Model Drugs (Part 1)

**Figure 1:**
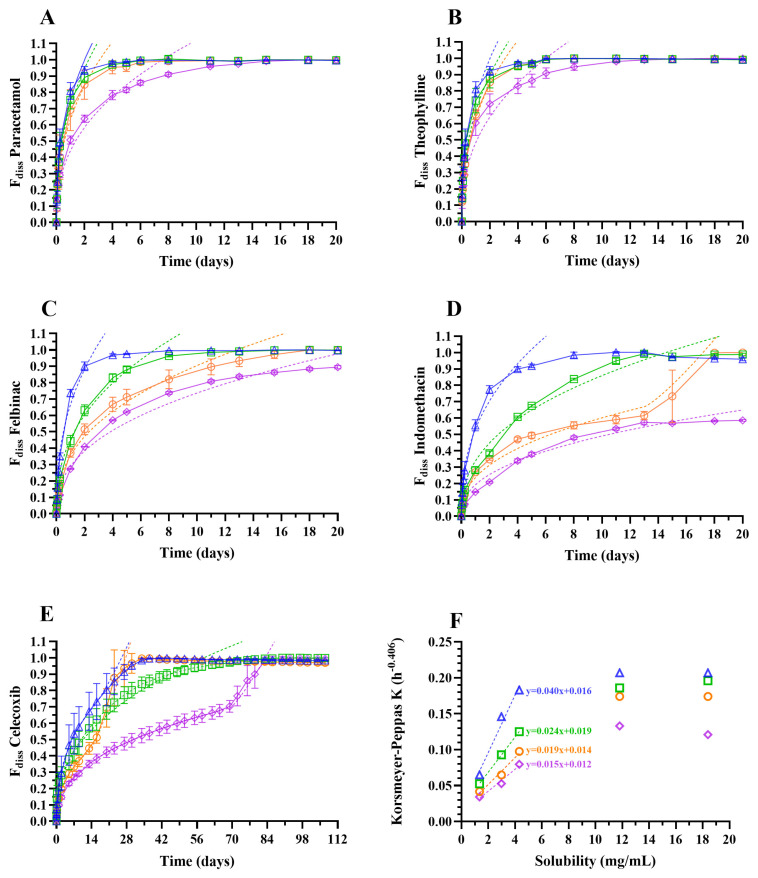
Panels (**A**–**E**): In vitro release (observed *F*_diss_)–time profiles of in situ gel (ISG) formulations 1 to 4 containing 10 mg/g of 5 model compounds, with solid lines representing mean ± SD (*N* = 4) and dotted lines indicating Korsmeyer–Peppas model fits. Panel (**F**): Relationship between Korsmeyer–Peppas release rate constants *K* and drug solubility in the IVR medium. Blue triangle: ISG formulation 1 (10 mg/g drug in PLGA50:50/NMP 30/70% (*w*/*w*)); green square: ISG formulation 2 (10 mg/g drug in PLGA85:50/NMP 30/70% (*w*/*w*)); orange circle: ISG formulation 3 (10 mg/g drug in PLGA50:50/NMP 40/60% (*w*/*w*)); purple diamond: ISG formulation 4 (10 mg/g drug in PLGA85:50/NMP 40/60% (*w*/*w*)); dotted lines represent the respective linear regression fits (*y* = *ax* + *b*).

Panels A to E in Figure 1 display the mean IVR profiles (observed *F*_diss_) and corresponding Korsmeyer–Peppas fits for ISG formulations 1 to 4 containing 10 mg/g paracetamol (A), theophylline (B), felbinac (C), indomethacin (D), or celecoxib (E) in 30% or 40% PLGA50:50 or PLGA85:15. Panel F illustrates the relationship between the Korsmeyer–Peppas release rate constants *K* and the solubility of the respective model drugs in their IVR medium.

Table 4 summarizes the IVR parameters and statistical comparisons for the observed IVR profiles and Table 5 provides the corresponding Korsmeyer–Peppas model parameters.

The mean %IVR observed within the first 48 h was notably high across all ISGs, ranging from 59% to 88% for paracetamol and theophylline, 41% to 90% for felbinac, 21% to 73% for indomethacin, and 15% to 30% for celecoxib. IVR profiles demonstrated that approximately 80% release was achieved within 1 to 5 days for paracetamol and theophylline ISGs, 1.5 to 11 days for felbinac ISGs, 3 to over 20 days for indomethacin ISGs, and 19 to 74 days for celecoxib ISGs, with a longer release duration for the 30% versus corresponding 40% PLGA formulations and for the PLGA50:50 versus corresponding PLGA85:15 formulations across compounds. Drug release exhibited biphasic patterns with an initial burst, except for indomethacin formulation 3 and celecoxib formulations 3 and 4, which showed triphasic profiles with a pronounced increase in drug release starting on day 15 or 16 for formulations 3 and on day 69 for celecoxib formulation 4. It should be noted that the IVR test for indomethacin was terminated after 20 days, before reaching complete drug release for formulation 4, and a potentially accelerated drug release phase at later time points cannot be ruled out.

For all five compounds, higher Korsmeyer–Peppas release rate constants *K* were observed for 30% versus the corresponding 40% PLGA formulations, as well as in PLGA50:50 compared to PLGA85:15 formulations. The release exponent *n* was below 0.5 for biphasic IVR profiles and the first two phases of the triphasic profiles, indicating a pseudo-Fickian diffusion-controlled drug release mechanism. Conversely, during the third phase of triphasic profiles, *n* exceeded 1, suggesting a super case II release mechanism.

As shown in Figure 1F, *K* increased with increasing solubility up to 5 mg/mL (felbinac, indomethacin, and celecoxib), with no significant effect above 12 mg/mL (paracetamol and theophylline). The observed increase in *K* with solubility was more pronounced for 30% versus 40% PLGA formulations and for PLGA50:50 versus PLGA85:15 formulations.

### 3.2. In Vitro Versus In Vivo Release for Celecoxib In Situ Gels (Part 2)

#### 3.2.1. Pharmacokinetic Study in Rats

**Figure 2:**
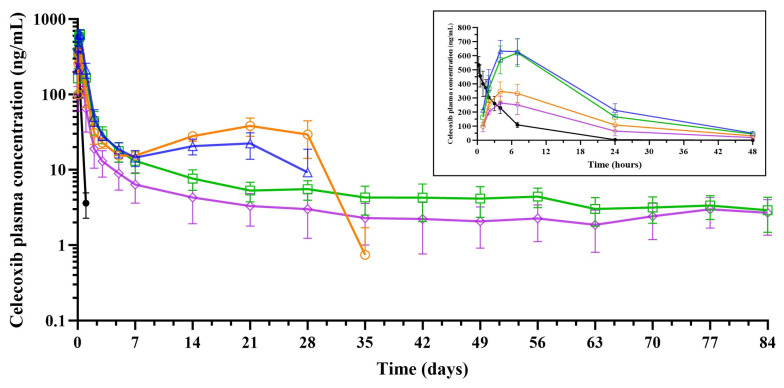
Plasma concentration–time profiles for celecoxib formulations 1 to 5 following injection in rat (mean ± SD for *N* = 4). Blue triangle: ISG formulation 1 (10 mg/g celecoxib in PLGA50:50/NMP 30/70% (*w*/*w*)) dosed subcutaneously (SC) at 10 mg/kg; green square: ISG formulation 2 (10 mg/g celecoxib in PLGA85:15/NMP 30/70% (*w*/*w*)) dosed SC at 10 mg/kg; orange circle: ISG formulation 3 (10 mg/g celecoxib in PLGA50:50/NMP 40/60% (*w*/*w*)) dosed SC at 10 mg/kg; purple diamond: ISG formulation 4 (10 mg/g celecoxib in PLGA85:15/NMP 40/60% (*w*/*w*)) dosed SC at 10 mg/kg; black dot: solution formulation 5 (1 mg/g celecoxib PEG400/water 50/50% (*v*/*v*)) dosed intravenously (IV) at 1 mg/kg.

Figure 2 presents the mean pharmacokinetic profiles of ISG formulations 1 to 4 administered at 10 mg/kg following dorsal SC injection in rats, along with the PEG400 solution formulation 5 dosed IV at 1 mg/kg. The corresponding pharmacokinetic parameters are summarized in Table 6.

Celecoxib plasma concentrations in rats were quantifiable up to 672 to 840 h (21 to 35 days) post-dose for PLGA50:50 formulations 1 and 3, and up to the last sampling time point of 2016 h (84 days) post-dose for PLGA85:15 formulations 2 and 4 (SC dose of 10 mg/kg). Quantifiable drug levels for the PEG400 solution formulation 5 were observed up to 24 h post-dose (IV dose of 1 mg/kg).

The mean pharmacokinetic profiles of ISG formulations 1 to 4 in plasma exhibited a pronounced peak between 4 and 7 h post-administration, followed by a rapid decline in plasma drug concentration. The IV PEG400 solution formulation 5 demonstrated a more rapid reduction in drug levels.

Notably, PLGA50:50 formulations 1 and 3 showed a secondary increase in celecoxib concentration starting at 168 h (7 days), reaching a peak at 504 h (21 days), which was higher for formulation 3 compared to formulation 1. Mean C_max_, AUC_48h_, AUC_168h_, and AUC_672h_ were higher for the 30% versus corresponding 40% PLGA formulations and for the PLGA50:50 versus corresponding PLGA85:15 formulations.

A two-compartment, IV-bolus input, first-order elimination pharmacokinetic model was fitted to the plasma concentration–time profiles following IV administration of PEG400 solution formulation 5 to characterize celecoxib distribution and elimination kinetics. This model served as the UIR function for the (de)convolution of the ISG drug release profiles (see Section 3.2.2), described by the equation *C*(*t*) = *A* × exp(−*αt*) + *B* × exp(−*βt*), where *A* = 1.78 ng/mL; *B* = 0.563 ng/mL; *α* = 0.201/h; *β* = 3.10/h.

Throughout the in vivo study, no significant clinical observations were noted in rats across groups 1 to 5. Postmortem examination revealed no residual ISG material in rats injected with formulation 1 or 3. For formulations 2 and 4, residual ISG size was similar among rats, averaging approximately 16 × 11 × 4 mm.

#### 3.2.2. In Vitro–In Vivo Comparison

**Figure 3:**
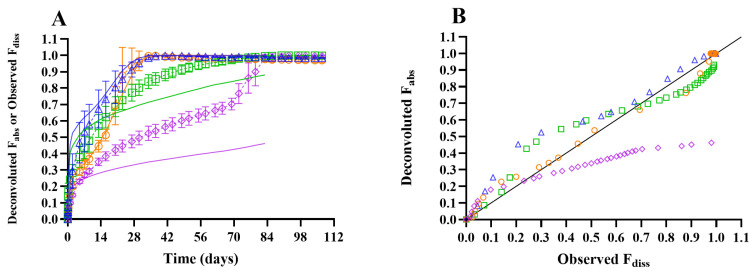
Panel (**A**): In vitro and in vivo drug release–time profiles for celecoxib ISG formulations 1 to 4, with dotted lines representing mean ± SD (*N* = 4) for fraction dissolved in vitro (observed *F*_diss_) and solid lines indicating mean (*N* = 4) fraction absorbed in rat obtained via deconvolution of observed pharmacokinetic profiles (deconvoluted *F*_abs_). Panel (**B**): Mean deconvoluted *F*_abs_ (*N* = 4) versus mean observed *F*_diss_ (*N* = 4). Blue triangle: in situ gel (ISG) formulation 1 (10 mg/g celecoxib in PLGA50:50/NMP 30/70% (*w*/*w*)); green square: ISG formulation 2 (10 mg/g celecoxib in PLGA85:15/NMP 30/70% (*w*/*w*)); orange circle: ISG formulation 3 (10 mg/g celecoxib in PLGA50:50/NMP 40/60% (*w*/*w*)); purple diamond: ISG formulation 4 (10 mg/g celecoxib in PLGA85:15/NMP 40/60% (*w*/*w*)); solid black line: line of unity (*y* = *x*). All ISG formulations were dosed subcutaneously at 10 mg/kg.

Figure 3A illustrates the in vitro fraction of celecoxib dissolved (observed *F*_diss_) and the in vivo fraction absorbed (deconvoluted *F*_abs_) over time for ISG formulations 1 to 4, with *F*_abs_ profiles exhibiting biphasic behavior for PLGA85:15 formulations and triphasic behavior for PLGA50:50 formulations.

In Figure 3B, the deconvoluted *F*_abs_ versus observed *F*_diss_ plot revealed that the correlation between in vitro *F*_diss_ and in vivo *F*_abs_ followed distinct phases: during the initial burst, *F*_abs_ exceeded *F*_diss_ for all formulations, whereas thereafter, *F*_abs_ closely aligned with *F*_diss_ for PLGA50:50 formulations and increased more gradually than *F*_diss_ for PLGA85:15 formulations.

Korsmeyer–Peppas parameters were fitted to the three distinct phases observed for celecoxib IVR profiles (observed *F*_diss_) and translated into in vivo drug release profiles (modeled *F*_abs_) by determining phase-specific scaling factors *A* for *K*, keeping *A* constant across formulations and phases where feasible (Table 7). During the initial burst release phase, scaling factors of 2.0 and 1.7 were applied to the 30% and 40% PLGA formulations, respectively. In the second phase, scaling factors of 0.72 and 0.45 were used for the PLGA85:15 and PLGA50:50 ISGs, respectively, with the scaling factor of 0.72 also applied to the final accelerated phase of the PLGA 50:50 formulations. Since the PLGA85:15 formulations did not exhibit a third phase in vivo and the scaling factor of 0.45 for the second phase indicated a significantly slower increase in *F*_abs_, the second IVR phase was employed for translation throughout the remainder of the in vivo *F*_abs_ profile.

**Figure 4:**
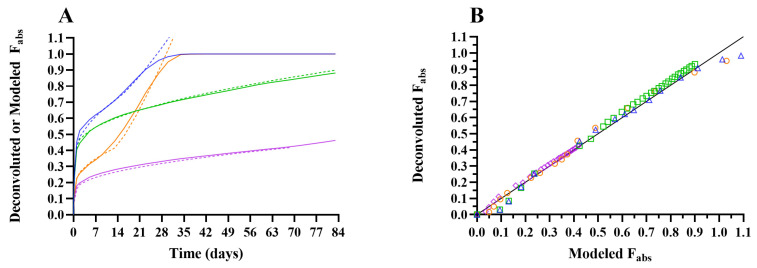
Panel (**A**): In vivo drug release–time profiles for celecoxib ISG formulations 1 to 4, with solid lines representing mean (*N* = 4) fraction absorbed in rat obtained via deconvolution of observed pharmacokinetic profiles (deconvoluted *F*_abs_) and dotted lines indicating the fraction absorbed, scaled from IVR Korsmeyer–Peppas model fits (modeled *F*_abs_). Panel (**B**): Mean deconvoluted *F*_abs_ (*N* = 4) versus modeled *F*_abs_. Blue triangle: in situ gel (ISG) formulation 1 (10 mg/g celecoxib in PLGA50:50/NMP 30/70% (*w*/*w*)); green square: ISG formulation 2 (10 mg/g celecoxib in PLGA85:15/NMP 30/70% (*w*/*w*)); orange circle: ISG formulation 3 (10 mg/g celecoxib in PLGA50:50/NMP 40/60% (*w*/*w*)); purple diamond: ISG formulation 4 (10 mg/g celecoxib in PLGA85:15/NMP 40/60% (*w*/*w*)); solid black line: line of unity (*y* = *x*). All ISG formulations were dosed subcutaneously at 10 mg/kg.

The deconvoluted and modeled *F*_abs_ profiles, presented as a function of time in Figure 4A and compared in Figure 4B, demonstrate a strong correlation, indicating that IVR data, when appropriately scaled, can reliably predict in vivo drug release behavior.

The modeled *F*_abs_ profiles, translated from in vitro Korsmeyer–Peppas fits, were convoluted using the UIR function obtained from IV formulation 5, and the corresponding pharmacokinetic parameters and internal prediction errors were calculated (summarized in Table 8). The observed C_max_ and partial AUCs, summarized in Table 6, were within 15% of the modeled pharmacokinetic parameters and mean absolute prediction errors were within 10% for all pharmacokinetic parameters. This demonstrates that the established point-to-point IVIVC effectively translated IVR data to in vivo pharmacokinetics across the four ISG formulations.

## 4. Discussion

### 4.1. In Vitro Release for In Situ Gels Containing Model Drugs

The IVR profiles were biphasic for the more soluble drugs paracetamol, theophylline, and felbinac, whereas the less soluble compounds indomethacin and celecoxib exhibited bi- or triphasic profiles. The biphasic IVR profiles were characterized by an early burst followed by a slower diffusion-controlled release, as evidenced by the Korsmeyer–Peppas exponents *n* below 0.5. The third phase observed for indomethacin and celecoxib formulations displayed *n* values exceeding 1, indicative of a super-case II mechanism predominantly driven by polymer erosion, relaxation, swelling, and/or dissolution processes. ISGs containing water-miscible solvents, such as NMP, are known to exhibit pronounced burst release due to a rapid phase inversion that leads to the formation of a solidified polymer shell with interconnected finger-like pores. During solvent/water exchange and pore formation in the shell, drug molecules located near the gel surface diffuse swiftly into the surrounding medium, resulting in a significant burst release [2,28,29,30,31]. Conversely, drug release from the core is more gradual, as the viscous polymer matrix impedes diffusivity, and progressive solidification occurs upon water penetration, creating a sponge-like porous inner structure [10,32,33]. During the diffusion-controlled phase, polymer erosion progressively accelerates, as hydrolytic degradation cleaves polymer chains into smaller fragments, thereby increasing porosity and facilitating enhanced water ingress. Ultimately, erosion in conjunction with polymer relaxation, dissolution, and swelling become the predominant mechanisms governing drug release, leading to a third, accelerated release phase [2,34]. This phase was only observed for the less soluble compounds indomethacin and celecoxib, starting around day 15 to 16 for PLGA50:50 formulations and around day 69 for PLGA85:15 formulations, a point at which drug release was already complete for the biphasic IVR profiles.

Across compounds, longer IVR release durations (time to 80% dissolved) and higher Korsmeyer–Peppas release rate constants *K* were observed for 30% versus 40% PLGA formulations, and for PLGA50:50 compared to PLGA85:15 formulations. This trend aligns with the expectation that increased polymer concentration and lipophilicity slow water ingress and phase inversion, resulting in a denser polymer matrix with a thicker shell that reduces burst release and prolongs drug release by limiting polymer erosion rate, swelling, and gel porosity [9,10,28,31,32,35].

When comparing IVR across compounds, minimal sustained release was observed for the sparingly soluble compounds paracetamol and theophylline with approximately 80% of the drug release within 1 to 5 days, whereas the slightly soluble compounds felbinac, indomethacin, and celecoxib exhibited longer release durations with 80% release occurring within 1.5 to 11 days, 3 to over 20 days, and 19 to 74 days, respectively. *K* increased with rising solubility up to 5 mg/mL, likely due to a steeper concentration gradient that enhanced diffusion rates for more soluble compounds. This effect was more pronounced in formulations with lower polymer lipophilicity or concentration, as these matrices facilitate water ingress, thereby amplifying the impact of drug solubility on release kinetics. Beyond a solubility of 12 mg/mL, no further change in *K* was detected, suggesting that the drug was fully dissolved within the polymer matrix early on and drug release was governed by the diffusional properties of the polymer matrix, such as porosity and tortuosity, rather than drug dissolution rate. It should be noted that the drug concentration in the ISGs was relatively low (10 mg/g), intentionally selected to ensure complete drug dissolution in the formulation matrix and enable direct comparison across compounds and formulations. At higher drug loadings, where partial suspension of the drug may occur, the release behavior differed substantially, and conclusions drawn here may not fully extrapolate to such conditions.

In summary, IVR rate, characterized by observed IVR parameters and Korsmeyer–Peppas release rate constants *K*, increased with decreasing polymer lipophilicity and concentration, as well as with increasing drug solubility in the IVR medium. Minimal sustained release up to 5 days was observed for the sparingly soluble compounds paracetamol and theophylline, whereas longer release durations—up to 11, over 20, and 74 days, respectively—were recorded for the slightly soluble compounds felbinac, indomethacin, and celecoxib.

### 4.2. In Vivo Versus In Vitro Release of Celecoxib In Situ Gels (Part 2)

Deconvoluted *F*_abs_ exceeded observed *F*_diss_ during initial burst release, with *K* scaling factors of 2.0 and 1.7 applied to translate IVR Korsmeyer–Peppas fits into *F*_abs_ for the 30% and 40% formulations, respectively. The higher in vivo burst may be explained by the limited body fluid available upon SC injection, leading a slower solidification of the polymer matrix compared to in vitro conditions, with higher diffusivity for the 30% formulations due to their lower viscosity relative to the 40% formulations [10,36]. Following the burst phase, deconvoluted *F*_abs_ closely aligned with observed *F*_diss_ for PLGA50:50 formulations with a *K* scaling factor of 0.72, whereas the lower scaling factor of 0.45 for PLGA85:15 formulations indicated a significantly slower drug release in vivo compared to in vitro. This discrepancy can be attributed to slower polymer erosion in vivo influenced by limited body fluid and mechanical pressure from surrounding tissue, which reduced ISG porosity, with the greatest divergence observed for more lipophilic polymers owing to slower water ingress [10,36]. The markedly slower polymer erosion for PLGA85:15 formulations in vivo likely accounts for the absence or delayed onset of a third accelerated release phase in vivo.

According to FDA’s IVIVC guidance, a level A IVIVC employs a single scaling factor throughout IVR profiles and across formulations and requires both internal and external validation [37]. In this study, however, shifts in the release mechanism and substantial differences in polymer concentration and grade precluded applying one universal IVIVC scaling factor to the celecoxib ISGs. Instead, a design-of-experiments approach identified phase- and property-specific scaling rules that produced robust translations of in vitro to in vivo drug release. The initial burst release could be scaled using the same *K* for ISGs with identical polymer concentration. Subsequent release phases could be scaled using a common *K* for ISGs with the same polymer grade, yielding modeled C_max_ and partial AUC values within 15% of the observed pharmacokinetic parameters. External validation is lacking because all four ISG formulations were used to build the IVIVC. Consequently, the developed IVIVC is appropriate for use for drug-development activities, but not for regulatory biowaivers. Literature supports the challenges encountered to build a traditional level A IVIVC for ISGs. For instance, Wang et al. developed a level A IVIVC for risperidone ISGs with slight variations in PLGA MW (17.8 to 21.7 kDa), using a time-scaled Makoid–Banakar dissolution model [14]. The model, however, failed validation because it markedly underpredicted C_max_ and could not be generalized to formulations with different PLGA L/G ratios. Similar limitations have been reported for level A IVIVCs of risperidone microspheres with minimal manufacturing variations, further emphasizing the need for adaptive scaling factors to reliably predict in vivo performance across diverse formulations [13,15].

We note that the mechanistic interpretations in this study were inferred from literature and from trends observed in the IVR and pharmacokinetic data and fitted model parameters. No dedicated mechanistic experiments were performed to directly confirm the hypothesized underlying processes. Targeted studies, such as time course molecular weight determinations, mass-loss measurements, and microscopy of the polymer matrix during release, would strengthen confidence in the proposed mechanisms and help to support the phase-specific scaling approach.

Despite these limitations, the presented IVIVC provides formulators with a practical tool for fine-tuning drug release through formulation optimization. By using a design-of-experiment strategy to identify phase-specific scaling factors for Korsmeyer–Peppas fits, the model successfully translated IVR data into in vivo release profiles across ISGs with varying polymer concentrations and grades.

## 5. Conclusions

This study demonstrates that IVR data for paracetamol, theophylline, felbinac, indomethacin, and celecoxib ISGs with 30% or 40% PLGA50:50 or PLGA85:15 exhibited sustained drug release, with release rates, modeled by Korsmeyer–Peppas equations, increasing as drug solubility in the IVR medium increased and as PLGA lipophilicity and polymer concentration decreased.

For celecoxib ISGs, a point-to-point IVIVC with phase-specific scaling factors for Korsmeyer–Peppas fits effectively translated IVR profiles to in vivo drug release following subcutaneous injection in rats.

## Figures and Tables

**Table 1:** Physicochemical properties of model compounds.

Model Compound	Aqueous Solubility at pH 7.4 ^a^ (mg/mL)	Solubility in PLGA50:50/NMP 40/60% (*w*/*w*) ^b^ (mg/g)	Solubility in PLGA85:15/NMP 40/60% (*w*/*w*) ^b^ (mg/g)	pKa ^c^	LogP ^d^	Molecular Weight ^c^ (g/mol)
Paracetamol	11.8	13.9	10.9	Strongest acidic: 7.82 Strongest basic: −0.78	−0.26	180
Theophylline	18.4	89.1	117	Strongest acidic: 9.5 Strongest basic: −4.4	0.51	151
Felbinac	4.31	59.4	56.9	Strongest acidic: 4.71 Strongest basic: NA	3.49	212
Indomethacin	2.98	163	160	Strongest acidic: 3.79 Strongest basic: −2.9	4.25	358
Celecoxib	0.00145 with 3%SLS: 1.35	57.4	53.9	Strongest acidic: 10.6 Strongest basic: −0.41	3.99	381

^a^ Experimentally determined by shaking an excess amount of the compound in 8 mL of 0.05 N phosphate buffer (pH 7.4) at 37 °C in an Innova incubator shaker (New Brunswick Scientific, Edison, NJ, USA) set to 100 rpm for 24 to 96 h. ^b^ Experimentally determined by shaking an excess amount of the compound in 3 g PLGA/NMP formulation at 25 °C in an Innova incubator shaker set to 100 rpm for 24 h. ^c^ Predicted from Chemaxon [25]. ^d^ Predicted from Aqueous logP Prediction System (ALOGPS) [25]. NA: Not applicable; NMP: N-methyl-2-pyrrolidone; PLGA: poly(D,L-lactide-co-glycolide); SLS: sodium lauryl sulfate.

**Table 2:** Formulations prepared for in vitro and in vivo assessments.

Formulation Type	Formulation Number/ Short Notation	Polymer Grade (L/G Ratio)	Solvent	Drug Concentration	Polymer/ Solvent Ratio	Formulation Density ^a^ (g/mL)	Assessments
ISG	1/30% PLGA50:50	PLGA50:50	NMP	10 mg/g	30/70% (*w*/*w*)	1.06	Part 1: IVR Part 2: celecoxib PK
ISG	2/30% PLGA85:15	PLGA85:15	NMP	10 mg/g	30/70% (*w*/*w*)	1.08	
ISG	3/40% PLGA50:50	PLGA50:50	NMP	10 mg/g	40/60% (*w*/*w*)	1.05	
ISG	4/40% PLGA85:15	PLGA85:15	NMP	10 mg/g	40/60% (*w*/*w*)	1.08	
Solution	5/PEG400 solution	PEG400	Water	1 mg/mL	70/30% (*v*/*v*)	NA	Part 2: celecoxib PK

^a^ Measured gravimetrically by weighing 1 mL of each ISG. ISG: in situ gel; IVR: in vitro release; L/G ratio: lactide/glycolide ratio; NA: not applicable; NMP: N-methyl-2-pyrrolidone; PEG: polyethylene glycol; PK: pharmacokinetics after subcutaneous injection in rat; PLGA: poly(D,L-lactide-co-glycolide).

**Table 3:** Pharmacokinetic study design for celecoxib ISGs in rats.

Group	*N*	Formulation Type	Formulation Number/ Short Notation	Dosing Route	Dose (mg/kg)	Dosing Volume (mL/kg)	Assessments
1	4	ISG	1/30% PLGA50:50	SC (Day 1)	10	1 g/density in g/mL	PK: 0–2016 h (84 days)
2	4	ISG	2/30% PLGA85:15	SC (Day 1)	10	1 g/density in g/mL	
3	4	ISG	3/40% PLGA50:50	SC (Day 1)	10	1 g/density in g/mL	
4	4	ISG	4/40% PLGA85:15	SC (Day 1)	10	1 g/density in g/mL	
5	4	Solution	5/PEG400 solution	IV (Day 1)	1	1	PK: 0–72 h (3 days)

Formulation 1: 10 mg/g celecoxib in PLGA50:50/NMP 30/70% (*w*/*w*); Formulation 2: 10 mg/g celecoxib in PLGA85:15/NMP 30/70% (*w*/*w*); Formulation 3: 10 mg/g celecoxib in PLGA50:50/NMP 40/60% (*w*/*w*); Formulation 4: 10 mg/g celecoxib in PLGA85:15/NMP 40/60% (*w*/*w*); Formulation 5: 1 mg/g celecoxib PEG400/water 50/50% (*v*/*v*); ISG: in situ gel; IV: intravenous injection; *N*: sample size; PK: pharmacokinetics after subcutaneous injection in rat; SC: subcutaneous injection.

**Table 4:** IVR parameters for ISG formulations containing model drugs.

Model Drug	Formulation Number/ Short Notation	*N*	%IVR at 48 h Mean (SD)	Time to 50% Dissolved (h) Mean (SD)	Time to 80% Dissolved (h) Mean (SD)	*f*2/*f*1
Paracetamol	1/30% PLGA50:50	4	86.9 (2.2)	7.58 (3.56)	26.7 (6.9)	1 vs. 2: 61/6 1 vs. 3: 36/22 1 vs. 4: 23/38 2 vs. 3: 43/12 2 vs. 4: 26/30 3 vs. 4: 35/20
	2/30% PLGA85:15	4	82.0 (3.1)	9.57 (4.13)	36.4 (8.9)	
	3/40% PLGA50:50	4	76.5 (6.2)	13.4 (6.8)	44.6 (20.8)	
	4/40% PLGA85:15	4	59.1 (1.9)	26.2 (3.4)	125 (8)	
	Ranking		1 > 2 > 3 ^a^ > 4	1 < 2 < 3 ^a^ < 4	1 < 2 < 3 ^a^ < 4	
Theophylline	1/30% PLGA50:50	4	87.7 (2.8)	7.82 (3.38)	29.1 (7.5)	1 vs. 2: 73/5 1 vs. 3: 51/18 1 vs. 4: 41/28 2 vs. 3: 60/10 2 vs. 4: 46/22 3 vs. 4: 53/13
	2/30% PLGA85:15	4	83.1 (5.7)	8.87 (4.05)	37.0 (13.8)	
	3/40% PLGA50:50	4	80.7 (6.2)	15.5 (1.6)	40.2 (15.5)	
	4/40% PLGA85:15	4	65.4 (3.4)	16.0 (7.1)	88.5 (29.2)	
	Ranking		1 > 2 > 3 ^a^ > 4	1 < 2 ^a^ < 3 < 4	1 < 2 < 3 ^a^ < 4	
Felbinac	1/30% PLGA50:50	4	90.4 (2.6)	12.8 (0.9)	32.8 (3.1)	1 vs. 2: 24/38 1 vs. 3: 19/48 1 vs. 4: 14/61 2 vs. 3: 37/17 2 vs. 4: 25/33 3 vs. 4: 37/15
	2/30% PLGA85:15	4	62.0 (3.2)	32.3 (4.4)	92.7 (7.0)	
	3/40% PLGA50:50	4	51.3 (5.2)	49.0 (13.0)	194 (75)	
	4/40% PLGA85:15	3	40.9 (0.1)	75.2 (0.7)	257 (12)	
	Ranking		1 ^a^ > 2 ^a^ > 3 > 4	1 ^a^ < 2 < 3 ^a^ < 4	1 ^a^ < 2 < 3 < 4	
Indomethacin	1/30% PLGA50:50	4	73.2 (3.0)	22.5 (3.0)	77.0 (9.6)	1 vs. 2: 18/49 1 vs. 3: 16/52 1 vs. 4: 11/68 2 vs. 3: 23/22 2 vs. 4: 18/34 3 vs. 4: 21/22
	2/30% PLGA85:15	4	32.7 (1.6)	93.1 (3.6)	271 (23)	
	3/40% PLGA50:50	4	32.9 (0.5)	186 (44)	389 (27)	
	4/40% PLGA85:15	4	21.0 (0.5)	219 (14)	>480	
	Ranking		1 ^a^ > 3 > 2 ^a^ > 4	1 ^a^ < 2 < 3 < 4	1 ^a^ < 2 ^a^ < 3 ^a^ < 4	
Celecoxib	1/30% PLGA50:50	4	29.5 (10.4)	161 (95)	456 (117)	1 vs. 2: 54/18 1 vs. 3: 43/26 1 vs. 4: 30/49 2 vs. 3: 51/23 2 vs. 4: 33/41 3 vs. 4: 41/32
	2/30% PLGA85:15	4	28.2 (5.2)	240 (69)	710 (120)	
	3/40% PLGA50:50	4	19.8 (1.2)	370 (43)	539 (89)	
	4/40% PLGA85:15	4	14.6 (1.1)	741 (136)	1787 (82)	
	Ranking		1 > 2 ^a^ > 3 ^a^ > 4	1 < 2 ^a^ < 3 ^a^ < 4	1 < 3 < 2 ^a^ < 4	

^a^ Significant statistical difference versus next ranked formulation, based on *p* value < 0.05 obtained via two-tailed, heteroscedastic *t*-test. Formulation 1: 10 mg/g drug in PLGA50:50/NMP 30/70% (*w*/*w*); Formulation 2: 10 mg/g drug in PLGA85:15/NMP 30/70% (*w*/*w*); Formulation 3: 10 mg/g drug in PLGA50:50/NMP 40/60% (*w*/*w*); Formulation 4: 10 mg/g drug in PLGA85:15/NMP 40/60% (*w*/*w*); *f*1: *f*1 difference factor; *f*2: *f*2 similarity factor; ISG: in situ gel; IVR: in vitro release: *N*: sample size; SD: standard deviation.

**Table 5:** In vitro release Korsmeyer–Peppas model parameters for ISG formulations containing model drugs.

Model Drug		Paracetamol			Theophylline			Felbinac			Indomethacin			Celecoxib		
Korsmeyer–Peppas Parameters		Time (h)	*K* (h^−n^)	*n*	Time (h)	*K* (h^−n^)	*n*	Time (h)	*K* (h^−n^)	*n*	Time (h)	*K* (h^−n^)	*n*	Time (h)	*K* (h^−n^)	*n*
1st and 2nd IVR phase	1/30% PLGA50:50	0–480	0.207	0.406	0–480	0.207	0.406	0–480	0.183	0.406	0–480	0.146	0.406	0–312	0.0652	0.406
	2/30% PLGA85:15		0.196			0.186			0.125		0–480	0.0928		0–2568	0.0527	
	3/40% PLGA50:50		0.174			0.174			0.0977		0–312	0.0649		0–312	0.0417	
	4/40% PLGA85:15		0.121			0.133			0.0796		0–480	0.0529		0–1656	0.0342	
3rd IVR phase	1/30% PLGA50:50	NA	NA	NA	NA	NA	NA	NA	NA	NA	NA	NA	NA	312–2568	0.30 × 10^−3^	1.22
	2/30% PLGA85:15										NA	NA	NA	NA	NA	NA
	3/40% PLGA50:50										312–480	1.20 × 10^−3^	1.22	312–2568	0.45 × 10^−3^	1.22
	4/40% PLGA85:15										NA	NA	NA	1656–2568	0.25 × 10^−3^	1.22
Pearson’s coefficient	1/30% PLGA50:50	0.957			0.957			0.976			0.978			0.968		
	2/30% PLGA85:15	0.963			0.962			0.992			0.996			0.988		
	3/40% PLGA50:50	0.954			0.982			0.990			0.971			0.977		
	4/40% PLGA85:15	0.964			0.963			0.990			0.992			0.991		

Formulation 1: 10 mg/g drug in PLGA50:50/NMP 30/70% (*w*/*w*); Formulation 2: 10 mg/g drug in PLGA85:15/NMP 30/70% (*w*/*w*); Formulation 3: 10 mg/g drug in PLGA50:50/NMP 40/60% (*w*/*w*); Formulation 4: 10 mg/g drug in PLGA85:15/NMP 40/60% (*w*/*w*); IVR: in vitro release; *K*: in vitro Korsmeyer–Peppas release rate constant; NA: Not applicable; *n*: in vitro Korsmeyer–Peppas release exponent.

**Table 6:** Pharmacokinetic parameters in rat following subcutaneous administration of celecoxib formulations 1 to 5.

Analyte		Celecoxib				
Species/Sex		Sprague Dawley Rat/Male				
Dosing Route		Subcutaneous				Intravenous
Formulation Type		ISG				Solution
Formulation Number		1	2	3	4	5
Dose (mg/kg)		10	10	10	10	1
PK parameter:						
*N*		4	4	4	4	4
C_0h_		NA	NA	NA	NA	626 (38)
C_max_ (ng/mL)	Mean (SD)	670 (80)	636 (83)	354 (59)	274 (56)	NA
t_max_ (h)	Min–Max	4.0–7.0	4.0–7.0	4.0–7.0	4.0–7.0	NA
t_last_ (h)	Min–Max	672–840	672–840	2016	2016	24
AUC_48h_ (ng·h/mL)	Mean (SD)	13,661 (995)	12,157 (1565)	7243 (1619)	5131 (1592)	2853 (340)
AUC_168h_ (ng·h/mL)	Mean (SD)	16,520 (1211)	14,998 (2099)	9540 (1788)	6406 (2061)	2854 (340)
AUC_672h_ (ng·h/mL)	Mean (SD)	25,710 (1640)	18,736 (2988)	24,406 (1749)	8465 (2778)	2854 (340)
AUC_last_ (ng·h/mL)	Mean (SD)	26,045 (2173)	23,909 (4863)	26,254 (3462)	11,640 (4188)	2836 (333)
AUC_∞_ (ng·h/mL)	Mean (SD)	NA	NA	NA	NA	2854 (340)
t_1/2_ (h)	Mean (SD)	NA	NA	NA	NA	3.34 (0.23)
Rank order C_max_		1 > 2 ^a^ > 3 ^a^ > 4				NA
Rank order AUC_48h_		1 > 2 ^a^ > 3 ^a^ > 4				
Rank order AUC_168h_		1 > 2 ^a^ > 3 ^a^ > 4				
Rank order AUC_672h_		1 > 3 ^a^ > 2 ^a^ > 4				

^a^ Significant statistical difference versus next ranked formulation, based on *p* value < 0.05 obtained via two-tailed, heteroscedastic *t*-test. AUC_xh_: area under the plasma concentration–time curve from time zero to x hours after dosing; C_0h_: plasma concentration at time zero after dosing; C_max_: maximal plasma concentration; ISG: in situ gel; formulation 1: 10 mg/g celecoxib in PLGA50:50/NMP 30/70% (*w*/*w*); formulation 2: 10 mg/g celecoxib in PLGA85:15/NMP 30/70% (*w*/*w*); formulation 3: 10 mg/g celecoxib in PLGA50:50/NMP 40/60% (*w*/*w*); formulation 4: 10 mg/g celecoxib in PLGA85:15/NMP 40/60% (*w*/*w*); Max: maximum; Min: minimum; *N*: sample size; NA: not applicable; PK: pharmacokinetic; SD: standard deviation; t_1/2_: half-life.

**Table 7:** Korsmeyer–Peppas fits for in vitro release (observed *F*_diss_) profiles of celecoxib formulations 1 to 4 and scaling factors used to translate *F*_diss_ profiles into in vivo release (modeled *F*_abs_) profiles following subcutaneous administration in rats.

Formulation Number/Short Notation	Korsmeyer–Peppas Fits for In Vitro Release (Observed *F*_diss_) Profiles									
	Initial Burst			Slower 2nd Phase			Accelerated 3rd Phase			Pearson’s Coefficient
	Time (h)	*K*	*n*	Time (h)	*K*	*n*	Time (h)	*K*	*n*	
1/30% PLGA50:50	0–24	0.0485	0.471	0–312	0.0125	0.616	312–2568	0.30 × 10^−3^	1.22	0.969
2/30% PLGA85:15		0.0485		0–2568	0.0190	0.521	NA	NA	NA	0.988
3/40% PLGA50:50		0.0308		0–312	9.12 × 10^−3^	0.616	312–2568	0.45 × 10^−3^	1.22	0.978
4/40% PLGA85:15		0.0220		0–1656	0.0127	0.521	1656–2568	0.25 × 10^−3^	1.22	0.991
**Formulation Number/Short Notation**	**Scaling Factors for Modeling In Vivo Drug Release (Modelled** *F* **_abs_) Profiles**									
	**Initial burst**			**Slower 2nd phase**			**Accelerated 3rd phase**			**Pearson’s** **coefficient**
	**Time (h)**	*A* **_1_**		**Time (h)**	*A* **_2_**		**Time (h)**	*A* **_3_**		
1/30% PLGA50:50	0–24	2.00		0–312	0.72		312–1656	0.72		0.997
2/30% PLGA85:15		2.00		0–2016	0.45		NA	NA		0.999
3/40% PLGA50:50		1.70		0–312	0.72		312–1656	0.72		0.994
4/40% PLGA85:15		1.70		0–2016	0.45		NA	NA		0.999

*A*: scaling factor translating Korsmeyer–Peppas release rate constants from in vitro to in vivo; *F*_abs_: fraction of celecoxib absorbed in vivo in rat; *F*_diss_: fraction of celecoxib dissolved in vitro; formulation 1: 10 mg/g celecoxib in PLGA50:50/NMP 30/70% (*w*/*w*); formulation 2: 10 mg/g celecoxib in PLGA85:15/NMP 30/70% (*w*/*w*); formulation 3: 10 mg/g celecoxib in PLGA50:50/NMP 40/60% (*w*/*w*); formulation 4: 10 mg/g celecoxib in PLGA85:15/NMP 40/60% (*w*/*w*); *K*: in vitro Korsmeyer–Peppas release rate constant; NA: not applicable; *n*: in vitro Korsmeyer–Peppas release exponent; Pearson’s coefficients were calculated for fitted versus observed *F*_diss_ and for modeled versus deconvoluted *F*_abs_.

**Table 8:** Pharmacokinetic parameters obtained via convolution of the modeled in vivo drug release (modeled *F*_abs_) profiles of celecoxib formulations 1 to 4 following subcutaneous administration in rats.

Formulation Number/Short Notation	PK Parameters Obtained via Convolution of Modeled In Vivo Drug Release (Modeled *F*_abs_) Profiles									
	C_max_		AUC_48h_		AUC_168h_		AUC_672h_		AUC_2016h_	
	Modeled	%PE	Modeled	%PE	Modeled	%PE	Modeled	%PE	Modeled	%PE
1/30% PLGA50:50	635	5	11,867	13	15,115	9	24,308	5	24,318	7
2/30% PLGA85:15	640	−1	11,569	5	13,359	11	16,705	11	21,576	10
3/40% PLGA50:50	346	2	6711	7	9108	5	21,278	13	24,641	6
4/40% PLGA85:15	245	11	4698	8	5882	8	8102	4	11,332	3
Mean Absolute %PE	NA	5	NA	8	NA	8	NA	8	NA	6

AUC_xh_: area under the plasma concentration–time curve from time zero to x hours after dosing; C_max_: maximal plasma concentration; *F*_abs_: fraction of celecoxib absorbed in vivo in rat; formulation 1: 10 mg/g celecoxib in PLGA50:50/NMP 30/70% (*w*/*w*); formulation 2: 10 mg/g celecoxib in PLGA85:15/NMP 30/70% (*w*/*w*); formulation 3: 10 mg/g celecoxib in PLGA50:50/NMP 40/60% (*w*/*w*); formulation 4: 10 mg/g celecoxib in PLGA85:15/NMP 40/60% (*w*/*w*); NA: not applicable; %PE: internal prediction error, calculated as [(Observed PK parameter − Modeled PK parameter)/Observed PK parameter] × 100; PK: pharmacokinetic.

## Data Availability

The data presented in this study are available on request from the corresponding author.

## Abbreviations

The following abbreviations are used in this manuscript:

ACN	Acetonitrile
ALOGPS	Aqueous logP Prediction System
AUC_xh_	Area under the plasma concentration–time curve from time zero to x hours after dosing
AUC_∞_	Area under the plasma concentration–time curve from time zero to infinity
BSA	Bovine serum albumin
C_0h_	Plasma concentration at time zero after dosing
C_max_	Maximal plasma concentration
DMSO	Dimethylsulfoxide
*F*_abs_	Fraction absorbed
*F*_diss_	Fraction dissolved
HPLC	High-performance liquid chromatography
ISG	In situ gel
IV	Intravenous
IVIVC	In vitro–in vivo correlation
IVR	In vitro release
K2.EDTA	Potassium ethylenediaminetetraacetic acid
*K*	In vitro Korsmeyer–Peppas release rate constant
LAI	Long-acting injectable
LC-MS/MS	Liquid chromatography–tandem mass spectrometry
L:G	Lactide/glycolide ratio
Max	Maximum
Min	Minimum
mPEG-PLA	Methoxy-poly(ethylene glycol)-co-poly(D,L-lactide)
MW	Molecular weight
*N*	Sample size
NMP	N-methyl-2-pyrrolidone
*n*	In vitro Korsmeyer–Peppas release exponent
PDA	Photodiode array
PK	Pharmacokinetic(s)
PLA	Poly(D,L-lactide)
PLGA	Poly(D,L-lactide-co-glycolide)
PLA-PEG-PLA	poly(D,L-lactide)-co-poly(ethylene glycol)-co-poly(D,L-lactide)
PVDF	Polyvinylidene fluoride
SD	Standard deviation
SLS	Sodium lauryl sulphate
t_1/2_	Half-life
TFA	Trifluoroacetic acid
UHPLC	Ultra-high-performance liquid chromatography
